# Mechanism of influenza A M2 transmembrane domain assembly in lipid membranes

**DOI:** 10.1038/srep11757

**Published:** 2015-07-20

**Authors:** Elka R. Georgieva, Peter P. Borbat, Haley D. Norman, Jack H. Freed

**Affiliations:** 1 Department of Chemistry and Chemical Biology, Cornell University, Ithaca, NY 14853; 2 National Biomedical Center for Advanced ESR Technology, Cornell University, Ithaca, NY 14853; 3 School of Engineering, Cornell University, Ithaca, NY 14853.

## Abstract

M2 from influenza A virus functions as an oligomeric proton channel essential for the viral cycle, hence it is a high-priority pharmacological target whose structure and functions require better understanding. We studied the mechanism of M2 transmembrane domain (M2TMD) assembly in lipid membranes by the powerful biophysical technique of double electron-electron resonance (DEER) spectroscopy. By varying the M2TMD-to-lipid molar ratio over a wide range from 1:18,800 to 1:160, we found that M2TMD exists as monomers, dimers, and tetramers whose relative populations shift to tetramers with the increase of peptide-to-lipid (P/L) molar ratio. Our results strongly support the tandem mechanism of M2 assembly that is monomers-to-dimer then dimers-to-tetramer, since tight dimers are abundant at small P/L’s, and thereafter they assemble as dimers of dimers in weaker tetramers. The stepwise mechanism found for a single-pass membrane protein oligomeric assembly should contribute to the knowledge of the association steps in membrane protein folding.

Influenza A M2 protein is a small, 97 amino acid, single-pass transmembrane protein, which is known to oligomerize forming a tetrameric proton-selective channel (Fig. 1A)^1^,^2^,^3^. This protein plays an essential role in viral adjustment and successful replication in the host cell; therefore it is a target for drug development^4^,^5^,^6^,^7^. The activation of M2 is pH dependent, and a single histidine residue in the transmembrane domain (TMD) of each protomer, H37, shuttles the proton through the channel by altering its protonation state; a tryptophan residue, W41, serves as the channel gate^8^,^9^. Furthermore, M2 activation by low pH and the following conductance are controlled by inter-subunit interactions between H37 and W41^10^,^11^.

The tetramer of the M2TMD segment spanning residues 22–46 was identified as the minimum unit required for proton conductance^12^. In lipid membranes and detergent solutions M2TMD assumes a highly helical secondary structure^13^,^14^,^15^. The high resolution structures of various M2TMD constructs, solved by solution and solid state NMR reveal several distinct conformations of M2TMD, which are all closed to different extents in the absence of any anti-influenza drug in lipid bilayers^11^,^16^,^17^ or with drugs rimantadine^18^ or amantadine^19^,^20^ bound to the protein. NMR studies, conducted on a broad range of membrane compositions and at different pH, indicated the high structural variability of M2, which is modulated by the bilayer properties and environmental acidity^21^,^22^. The open state of detergent-reconstituted M2TMD at low pH of 5.3 was solved by x-ray crystallography^23^. This large body of structural information significantly advanced the understanding of the proton conductance mechanism and its channel inhibition^11^,^24^,^25^.

Another important aspect toward our understanding and controlling the function of M2 is to learn the mechanism of its assembly into a functional tetramer. In the viral envelope of influenza A, M2 is a minor component represented by some 10–20 molecules per virion^26^,^27^ that is just by 2–5 assembled tetramers. However, most of the *in vitro* structural studies on fully assembled M2TMD tetramers were conducted at peptide-to-lipid molar ratios exceeding 1:100 or even 1:50, thereby mimicking conditions of high M2 abundance. Hence, the pathway of M2 self-association and its forms under native or close to native conditions are not yet well understood. It has been proposed, based on a variety of biophysical and biochemical studies, that protein-to-lipid (detergent) molar ratio and lipid bilayer thickness strongly affect the thermodynamics and kinetics of M2TMD monomers self-association^14^,^15^,^28^,^29^,^30^. Analytical ultracentrifugation experiments on dodecylphosphocholine (DPC) micelle-bound M2TMD (residues 19–46) suggested monomer-tetramer equilibrium^28^, which was further supported by the study of thiol-disulfide equilibrium of the same peptide in detergent^29^ and lipid bilayers^15^. In the latter studies^15^,^29^, disulfide cross-linked M2TMD dimers were generated, which then assembled in tetramers. However, no significance was attributed to dimer formation in those studies. On the other hand, when expressed in mammalian cells, M2 shows oligomeric polydispersity and one of the detected species is a dimer^31^. Furthermore, recently the M2 dimer was proposed as the minimal proton-conducting unit, based on experiments on the full-length protein residing in the plasma membrane of CHO-K1 cells^32^. Hence, there is a gap in understanding the mechanism of M2 monomer assembly into a functional channel and the role of other oligomeric states.

To contribute to the understanding of this important issue, we employed pulse dipolar ESR distance measurements (DEER spectroscopy) with spin-labeling^33^,^34^,^35^,^36^ on the M2TMD_21–49_ peptide, which contains M2TMD and its N- and C-terminal juxtamembrane residues, with a spin-labeled cysteine residue L46C (Fig. 1B). DEER is well positioned to study protein self-aggregation and elucidation of protein oligomeric states, since the time-domain dipolar signal in DEER depends on the number of associated spin-labeled monomers^37^,^38^,^39^,^40^,^41^.

Based on a series of measurements in DOPC:POPS (1,2-dioleoyl-*sn*-glycero-3-phosphocholine: 1-palmitoyl-2-oleoyl-*sn*-glycero-3-phospho-L-serine) membranes by varying the peptide-to-lipid (P/L) molar ratio by a greater than a factor of 100 from 1:18,800 to 1:160, we determined that the M2TMD_21–49_ monomer oligomerizes into dimers and then the dimers form dimer-of-dimers, that is an M2 tetramer. We found that M2 assembly responds to the environmental pH by a shift in equilibria but not by any change of the mechanism, based on the results from experiments conducted at pH 5.5 and pH 8. The results for detergent, β-DDM (*n*-Dodecyl β-D-maltoside) encompassing the 1:6,000 to 1:10 (i.e. a factor of 600) range of peptide-to-detergent molar ratios also support this pathway of self-assembly, but the results are less obvious. This emphasizes the significance of bilayer properties for M2 assembly and the role of stabilization of its functionally relevant forms. Thus, we provide strong evidence that dimer formation is a critical step in M2 self-association. This study on M2TMD was enabled as a result of methodologically advancing the application of high-sensitivity DEER spectroscopy to characterize a complex equilibrium of integral membrane protein oligomers thereby contributing to our general understanding of self-association as a step in membrane protein folding.

## Results

### The assembly of M2TMD_21–49_ depends strongly on the peptide-to-lipid molar ratio (P/L), and to a much lesser extent on pH

**Figure 1:**
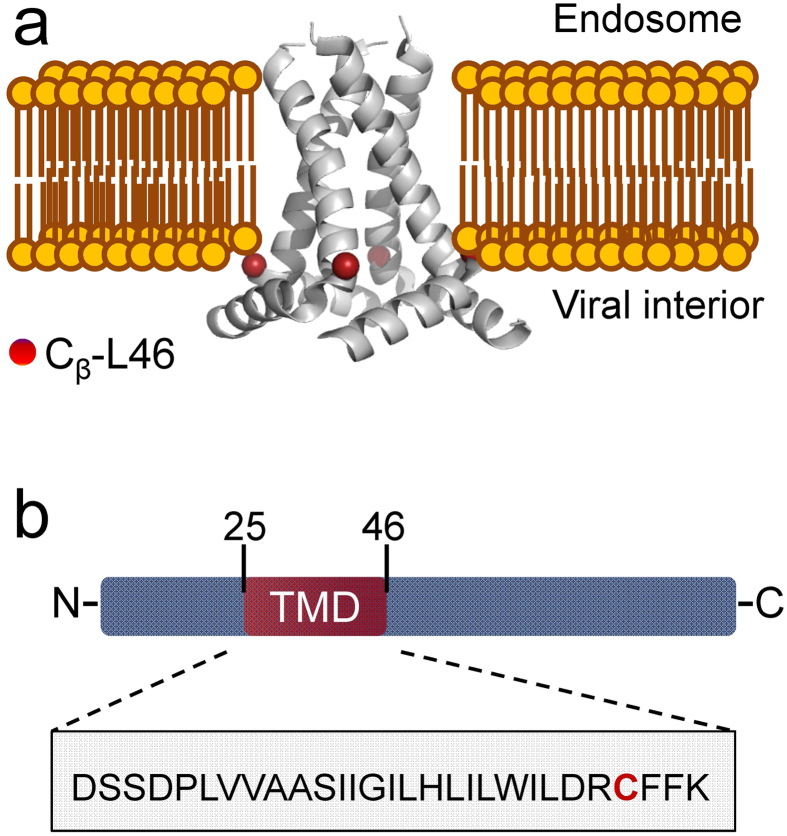
A glimpse at Influenza A M2. **a** In the structure of M2 tetramer one recognizes the transmembrane domain and C-terminal amphipathic helix as determined by Sharma *et al.*^11^ by solid state NMR (PDB ID: 2L0J). The surrounding lipid bilayer is added for clarity. The C_β_-atom of residue L46, mutated to cysteine for spin-labeling, is shown as red sphere in each of four protomers of M2. **b** Diagram representing the full length M2 shows the position of the transmembrane domain (TMD) formed by the residues 25–46 colored in dark red. The amino acid sequence of M2TMD_21–49_ used in this study is emphasized with L46C residue colored in red.

Recently, we reported preliminary results that M2TMD_21-49_ does not completely assemble into tetramers in lipid membranes of different compositions and peptide-to-lipid molar ratio of 1:500, but most likely exists as monomers, dimers and tetramers^42^. This motivates us to investigate in greater detail how this M2 construct oligomerizes to form functional channels. We studied the self-association of spin-labeled M2TMD_21–49_ at position L46C in lipid membranes of DOPC:POPS at 85:15% molar ratio. Including PS lipid is relevant to the native influenza virus membrane composition^43^. Although it was reported that M2TMD assembles in tetrameric channels under a broad range of pH conditions^21^,^22^, other studies find that either high pH^28^ or low pH^32^ is more favorable for tetramer formation. Therefore, we conducted our experiments on peptide/lipid systems at two different pH’s 5.5 and 8. We selected the residue L46C for spin-labeling, since it is positioned at the lipid-water interface in the C-terminal of M2TMD, just before the C-terminal amphipathic helix of full-length M2, as shown in Fig. 1. Furthermore, a previous study by continuous wave ESR (cw*-*ESR) did not report any perturbations due to spin-labeling and the results demonstrated that among several spin-labeled residues in M2, L46C follows the general pattern of structural transitions upon pH variations^44^. Thus, we considered the L46C residue as a suitable reporter on the assembly of M2TMD.

**Figure 2:**
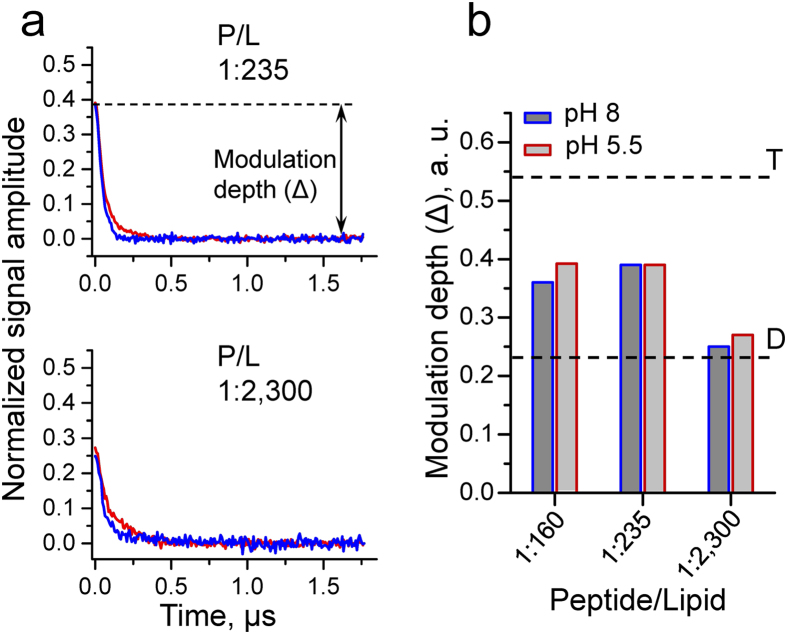
DEER data for M2TMD_21–49_ spin-labeled at position L46C in DOPC:POPS (85:15% molar ratio) membranes recorded at pH 5.5 (in red) and pH 8 (in blue). **a** Background corrected and normalized (Methods, Supplementary Figs. 1 and 7) time-domain DEER data for P/L molar ratios of 1:235 and 1:2,300. The DEER modulation depth (Δ) is indicated. **b** DEER modulation depths for P/L 1:160, 1:253 and 1:2,300 are visualized as bar plots. The margins of error due to experimental uncertainties and limitations of data analysis were found to be within ± 5%. The expected values for DEER modulation depth of 0.23 and 0.54 for dimer (D) and tetramer (T), respectively, are indicated by dashed lines. However, it is recognized that for a tetramer the theoretical maximum of 0.54 would be unlikely due to slightly less than unity spin-labeling efficiency and effects caused by uniformity of the magnetic component, *B*_1_, of microwave field over the sample in the resonator (Methods).

Based on the fact that the DEER signal depends on the number of coupled electron spins in a known quantitative way^34^,^36^,^38^,^40^,^41^,^45^, we investigated the M2TMD_21–49_ association by measuring the modulation depth of the time-domain DEER signals from spin-labeled residue L46C in the course of varying the P/L at two values of pH. The time-domain signal in DEER reports on the number of interacting electron spins in a small group of spins^34^,^36^,^38^,^40^,^41^,^45^, based on the following signal modifying properties: the DEER signal modulation depth (Δ), which is defined as Δ(*p*) = 1– DEER(t = ∞)/DEER(t = 0) (Fig. 2, Supplementary Fig. 1), where *p* is the fraction of spins flipped by the pump π-pulse, strongly depends on the number of spins, *N*, participating in the dipolar interaction as Δ(*p*,*N*) = [1 – (1 – *p*)^*N*−1^]^37^,^38^,^39^,^40^,^41^. Note that for a dimer Δ(*p,*2) = *p*. Thus, for an oligomer of higher order than a dimer, the modulation depth will be greater than *p*. Under our experimental conditions for doubly spin-labeled biomacromolecules, or dimers comprised of singly labeled monomers, *p* is 0.23^46^, for a 32 ns pump pulse and for a fully assembled tetramer the ideal-case calculated value of Δ(*p*, 4) is 0.54. Since Δ(*p*, *N*) also depends on spin labeling efficiency, *f*, in order to ensure accurate and reproducible measurements, it is desirable to obtain labeling efficiency close to unity in order not to unnecessarily complicate the data analysis. In this work close to 100% peptide spin-labeling was achieved, as confirmed by Mass Spectroscopy (Supplementary Fig. 2). Also the presence of unreacted free spin label can decrease the apparent Δ(*p*, *N*); but by means of careful purification no free spin-label was detected in the samples as inferred from the cw-ESR spectra (Supplementary Fig. 3). The efficiency of M2TMD_21-49_ assembly at different pH’s could thus be quantitatively assessed by measuring the modulation depth of corresponding DEER signals, taking into account additional experimental factors that affects the signal.

**Figure 3:**
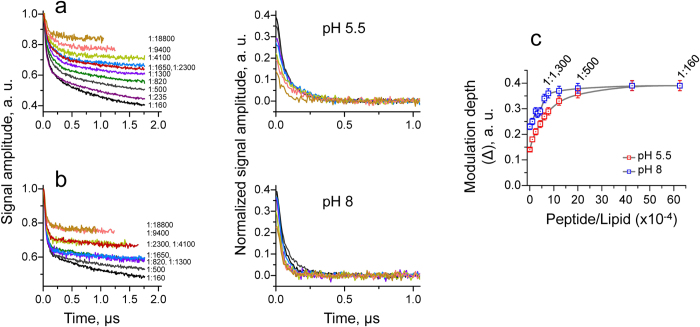
Experimental time-domain DEER data for M2TMD_21–49_ spin-labeled at position L46C. The protein was reconstituted into DOPC:POPS (85:15% molar ratio) at pH 5.5 **a**, and pH 8 **b**. Normalized raw data are compiled in the left **a** and **b** panels with P/L ratios given for each signal. Background-corrected data are plotted in the right **a** and **b** panels such as to have asymptotic value of zero and are normalized to have the amplitude at zero time equal to the modulation depth. (cf. Supplementary Fig. 1). The data for P/L’s 1:18,800, 1:9,400, 1:4,100, 1:2,300 1:1,650, 1:1,300, 1:820 1:500, 1:235 (only for pH 5.5) and 1:160 with progressively increasing modulation depths (Δ) values are shown. The colors of raw signals (left panels) match the colors of background corrected signals (right panels). **c** The modulation depth values plotted for samples spanning the whole range of P/L’s, namely 1:18,800, 1:9,400, 1:4,100, 1:2,300, 1:1,650, 1:1,300, 1:820, 1:500, 1:235, and 1:160, for both pH’s 5.5 and 8, plus an extra point 1:3,170 available for pH 8. The error bars correspond to the estimated error margins of ± 5% (cf. note in Fig. 1 caption).

The background corrected and normalized time-domain DEER data for M2TMD_21–49_ reconstituted in DOPC:POPS membranes at P/L ratios of 1:235 and 1:2,300, at pH 5.5 and pH 8 are plotted in Fig. 2a. The modulation depths estimated from time-domain DEER signals at P/L of 1:160, 1:235 and 1:2,300 at both pH’s are shown in Fig. 2b. Thus, we noticed that in these cases the DEER modulation depths do not correspond to a fully assembled tetramer, since the values were significantly smaller than 0.54 (Fig. 2). One would reasonably expect to observe slightly reduced values of Δ(*p*, *N*) for a tetramer even for *f* close to 100% due to the system and experiment properties; also the possible presence of a subensemble of channels with inter-label distances shorter than ~2 nm would introduce a slight reduction of Δ(*p*, *N*). It was confirmed by cw-ESR and double-quantum coherence ESR spectroscopies that this short-distance subensemble is indeed a very small fraction (cf. Methods). However, these possibilities would contribute a constant factor to the DEER modulation depth, which cannot explain the result that the modulation depths varied so significantly depending on the P/L ratio. A decrease of about 32% in Δ(*p*, *N*) was observed for a P/L of 1:2,300 with respect to the higher P/L of 1:160 and 1:235 for both pH 5.5 and 8 (Fig. 2), suggesting that the P/L does play a role in the peptide assembly. To better understand this, we conducted DEER experiments on a series of samples with P/L ratios spanning broad range from 1:18,800 up to 1:160. This produced a monotonic increase in DEER modulation depth, with a factor of change of ca. 3 for pH 5.5 and ca. 2 for pH 8 measured over the whole range of P/L ratios (Fig. 3a–c). Notably, in the case of pH 5.5 the modulation depth value of 0.14 at very low P/L of 1:18,800, was considerably smaller than 0.23, expected from a system of two coupled nitroxides. Also, the modulation depth value of ca. 0.19 at the same P/L but pH 8 was close to that expected from dimer. In both cases, however, these values were significantly less than those estimated for a fully assembled M2TMD tetramer, i.e. Δ(*p*, 4) = 0.54. On the other hand, at the highest P/L these values were about 0.4, which is much closer to 0.54 expected for the tetrameric form. So far, the variations in DEER modulation depth, mostly falling within the range between those of dimer and tetramer, point to polydispersity of M2TMD_21–49_ oligomeric species. Strikingly, the growth of modulation depth progressively with P/L suggests that P/L determines the relative population of each of these species.

Our observations here are generally consistent with the previously reported more efficient M2TMD self-association in DPC micelles and phospholipid bilayers, upon increase of P/D and P/L ratios^15^,^28^,^29^. However, our quantitative results based on the concentration profile of DEER modulation depths unequivocally support a more complex equilibrium that does require the presence of dimers, which is different from the previously crafted simpler picture of monomer-tetramer reversible transition^15^,^28^,^29^.

Interestingly, we see a difference in the DEER modulation depth development with P/L when we compare the data for pHs 5.5 and 8, (Fig. 3). The difference between the smallest values of modulation depths observed at P/L of 1:18,800 was as large as ca. 0.1. This trend is maintained over the whole range of P/L studied (Fig. 3a–c). These results point to stronger M2TMD_21–49_ self-association at pH 8 than at pH 5.5.

**Figure 4:**
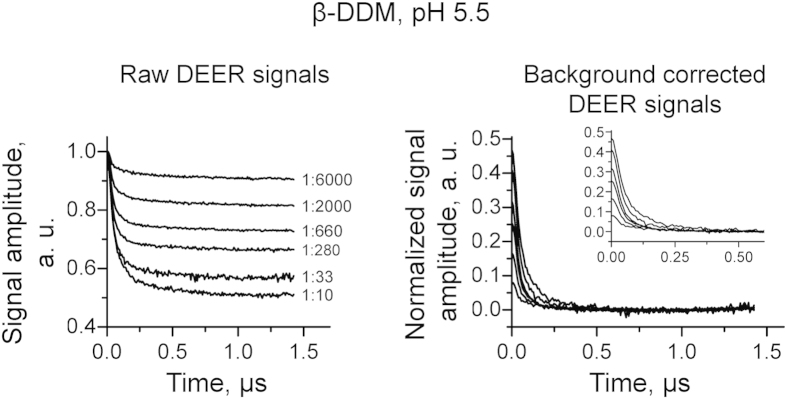
Experimental time-domain DEER signals for M2TMD_21–49_ spin-labeled at position L46C and reconstituted in β-DDM at pH 5.5. Raw data are shown on the left with P/D ratios provided for each signal. Background corrected and normalized data are on the right.

To complement our results in lipid, we carried out similar experiments in detergent (β-DDM)-reconstituted peptide at pH 5.5. However, in order to record DEER signals with maximal modulation depth range of change, similar to that for lipid-reconstituted peptide, it was necessary to shift the range of P/D to larger ratios and a somewhat wider range from 1:6,000 up to 1:10 (Fig. 4), which unambiguously indicates a different equilibrium for M2TMD_21–49_ self-association in lipid vs. detergent. But, in detergent as in lipid, we observed modulation depths increasing from ca. 0.08 to 0.46, or by a factor of ca. 6, indicating the possibility of multiple oligomeric species with P/D-dependent populations.

Furthermore, the inter-spin distances reconstructed from the time-domain DEER signals were, overall, in agreement with those predicted based on NMR and X-ray crystallography for spin-labeled residue L46C (Supplementary Fig. 4).

### Model of M2TMD_21-49_ self-association in DOPC/POPS bilayers

**Figure 5:**
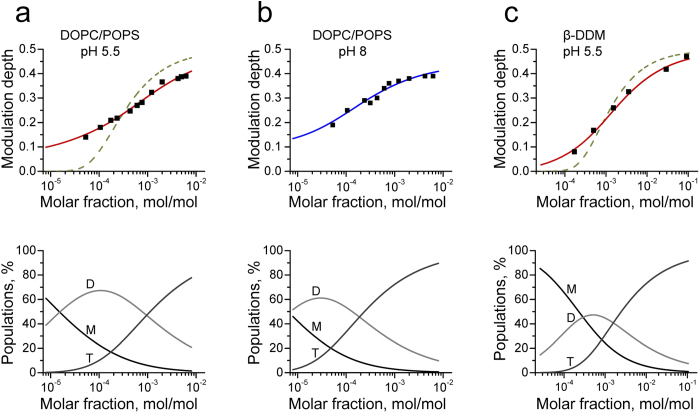
Concentration profiles of M2TMD_21–49_ monomers, dimers and tetramers as a function of P/L and P/D molar ratios. DEER signal modulation depth concentration profiles (filled squares) are plotted in the top row vs. M2 molar fraction for DOPC/POPS lipid mixture in **a** and **b** and β-DDM in **c**. The fits of these experimental data to the equilibrium model based on tandem momomer↔dimer↔tetramer vs. that for monomer↔tetramer model (for lipid and detergent at pH 5.5) are plotted in solid red and dashed green lines, respectively in **a** and **c** upper panels. Clearly, the tandem model is necessary to describe the M2TMD assembly pathway. Respective equilibrium constants, *k*_2d_, *k*_4d_ for the tandem model are 15·10^−6^ MF and 448·10^−6^ MF, pointing to strong binding for dimers but relatively weaker bound tetramers. In β-DDM these constants both are weak and close to each other, being 264·10^−6^ and 644·10^−6^ MF. Concentration profiles of populations for M2TMD_21–49_ monomers (M), dimers (D), and tetramers (T) are plotted in **a** and **b** lower panels for DOPC/POPS lipid membranes at pH’s 5.5 and 8, respectively, and for β-DDM in **c** lower panel. The populations of each fraction are expressed as M2 percentages of total M2TMD_21–49_ monomer concentration, C_M2TMD_ = *C*_M_ + *C*_D_ + *C*_T_. Here *C*_M2TMD_ is expressed as M2 molar fraction, 1/(1+A/P), with A/P is amphiphile-to-protein molar ratio.

The obtained modulation depth values in DOPC/POPS at pH’s 5.5 and 8 are plotted in Fig. 5a,b upper panels, as a function of M2TMD_21–49_ mole fraction (MF), which is moles peptide/(moles peptide + moles lipid or detergent). The same type of data for the peptide in β-DDM at pH 5.5 are shown in Fig. 5c upper panel. The equilibrium parameters of M2TMD_21–49_ self-association in lipid membranes can be directly determined by analyzing the growth of DEER modulation depth as a function of P/L. A similar approach proved successful in a study of lipoxygenase-ligand interaction^47^.

In the case of lipid (Fig. 5a,b upper panels), qualitatively we see two distinct ranges characterized by different slopes in the DEER modulation growth, which corresponds to MFs from ca. 5.3·10^−5^ to 6·10^−4^ (1:18,800 to 1:1,650 P/L molar ratio) and from ca. 6·10^−4^ to 6.2·10^−3^ (1:1,650 to 1:160 P/L molar ratio). An attempt to fit these results for both pH’s to a model of monomer-tetramer equilibrium^15^,^28^,^29^ over the wide concentration range of our results proved highly unsuccessful. This required us to improve the model by making the assumption of a tandem equilibrium model, 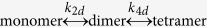. This two-stage model can be fit in terms of monomer-to-dimer (2M↔D) and dimer-to-tetramer (2D↔T) equilibria defined by the dissociation constants *k*_2d_ and *k*_4d_, respectively. This model of consecutive self-association agrees very well with our experimental data, which we fitted to the respective set of equilibrium equations. The DEER modulation depth serves as a response function to the equilibrium state, wherein a tetramer contributes more than a dimer. Our assumption is also based on the reported literature data where for wild type M2 and mutants lacking all or some of native cysteine, produced in virions grown in embrionated eggs, only strong evidence about M2 monomers, dimers and tetramers was obtained^48^. Of course, we cannot completely rule out the formation of a minor population of other, possibly transient, oligomeric species, such as M2TMD_21–49_ trimers, which however would be difficult to quantify. It would be unreasonable to believe that proteins that assemble as strong dimers, and only at sufficiently high concentrations start to assemble into tetramers would ever exist to any significant extent as trimers, since monomers are only present as a minor fraction at the onset of tetramer assembly. The existence of trimers could also be ruled out with a high level of confidence, since they were not reported by any other spectroscopic techniques, particularly NMR.

Thus, we can write for the modulation depth the expression that directly follows from the analysis of DEER data in pure and mixed multiple-spin systems^38^:


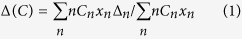


Here *C*_*n*_ are the concentrations of components with nominal number of spins, *n*, Δ_*n*_ are their respective modulation depths^38^ and *x*_*n*_ represent the effects of phase relaxation, but more generally *x*_*n*_ should combine the effects of relaxation, spin-labeling efficiency, *f*, and some variations in intensity based on the presence of short distances and orientational correlations. Indeed, the variations of *x*_*n*_-factor due to all above reasons in our case are quite small, based on the observed close relaxation properties of nitroxide-labeled M2TMD_21-49_ under all conditions, very close to unity *f*, and presumably negligible population of spin-labels at short distances (less than 2 nm) due to significant flexibility of nitroxide side-chain; correlations were found insignificant even for rigid polyradicals^38^. Below we will use notation *C*_M_, *C*_D_ and *C*_T_ for the concentration of M2TMD_21-49_ forming monomeric, dimeric, and tetrameric species. By solving the system of algebraic equation, describing 2M↔D and 2D↔T equilibria and the material balance given by *C*_M_ + 2*C*_D_ + 4*C*_T_ = *C*, where *C* is the total M2TMD_21–49_ concentration (expressed as its mole fraction), and then calculating the response function Δ(*C*), according to Eq. (1), we can compute the response Δ(*C*). The response then can be fitted to the respective experimental data by non-linear least squares optimization to obtain *k*_2d_, *k*_4d_, and concentration profiles for all species.

Thus, this two-stage model represented our experimental data well for lipid reconstituted M2TMD_21–49_, and based on this model we were able to characterize with a good accuracy the equilibria 2M↔D and 2D↔T (Fig. 5a,b upper panels). In the case of pH 5.5, we obtained *k*_2d_ of 15·10^−6^ MF (P/L ~ 1:65,000) and *k*_4d_ of 448·10^−6^ MF (P/L of 1:2,230). At pH 8 these constants were *k*_2d_ = 7.10^−6^ (P/L ~ 1:142,800) and *k*_4d_ = 86.10^−6^ (P/L ~ 1:11,600), shifted to even lower P/L compared to pH 5.5. Indeed, it was difficult to obtain from the fittings the *k*_2d_ value for the case of pH 8, because of the closeness to 0.23 (value for dimer) of DEER modulation depths for the lowest P/L we were able to employ, therefore the conditions of prevalent monomers were unreachable. The contribution of monomers is marginal at pH 8. The minimal stable oligomeric form of M2TMD_21–49_ is definitely a dimer. This suggests that at basic pH 8 dimers are more stable than those at acidic pH of 5.5. Thus, our thorough analysis of DEER data for both pH’s of 5.5 and 8 provides unambiguously evidence about the formation of tight dimers at very low P/L ratios, which at sufficiently high P/L ratios assemble into a dimer-of-dimers, that is weaker tetramer.

In detergent at pH 5.5, both *k*_2d_ and *k*_4d_ values were 264·10^−6^ MF (P/D ~ 1:3,800) and 644·10^−6^ MF (P/D of 1:1,555), respectively, which is about an order of magnitude greater than *k*_2d_ for lipid indicating the lesser stability of the M2TMD_21–49_ assembly in detergent relative to lipid. Also, both equilibrium constants in detergent are of the same order of magnitude, so that equilibrium could be roughly approximated by a monomer-tetramer equilibrium, however the fit to this model is still rather poor (Fig. 5c upper panel) compared to the dimer-based model. Figure 5a–c lower panels show the population profiles of M2TMD_21–49_ participating in monomers, dimers, and tetramers plotted as functions of M2TMD_21–49_ MF in lipid and detergent, respectively. The profiles rendered as percentages of total MF reflect the two-stage equilibrium kinetics, 2M↔D and 2D↔T, and are computed based on the parameters obtained by the fittings (Fig. 5a–c upper panels). In lipid, only under the very low MF of less than 10^−5^ M2TMD_21–49_ becomes noticeably monomeric. Notably, in detergent M2TMD_21–49_ is predominantly monomeric (>50%) at MFs as large as 10^−4^, and dimers efficiently assemble at MFs that are more than at least an order of magnitude higher than those for dimers in lipid. This suggests that the lipid bilayer is a strong determinant of M2 assembly and stability.

## Discussion

We studied the mechanism of assembly of M2TMD_21–49_, a peptide containing residues 21–49 of the M2 transmembrane domain with its N- and C-terminal juxtamembrane residues (Fig. 1b). We conducted our experiments on peptide residing in either lipid membranes of DOPC/POPS at 85:15% molar ratio or detergent, β-DDM. The lipid composition was selected to closely mimic the native environment: The 26.5–27 Å thickness of the DOPC/POPS bilayer^49^ satisfies the hydrophobic match requirements for M2TMD, which contains 19 amino acids forming a ca. 28.5 Å long helix. Furthermore, PS is a physiological component of influenza viral membrane^43^. In addition, no cholesterol affinity was reported for M2 under native conditions^50^. Remarkably, it was found that in the viral membrane M2 is a low-abundance protein, which constitutes about 3% of the envelope proteins represented by only 16–20 M2 polypeptides per virion^26^,^27^. This is an equivalent to just 4 to 5 fully assembled tetrameric proton channels. Based on these numbers, we can estimate the molar ratio of M2 polypeptides to total lipid in a typical virion. Assuming that the lipid headgroup occupies ~50 Å^2^ surface area, a rough estimate shows that each of the leaflets of a spherical bilayer of 1000 Å average diameter (such as of a typical virion^51^) contains about 6.3 × 10^4^ lipid molecules leading to an M2-to-lipid molar ratio of ~1:6,280. However, one should take into account the presence of hemagglutinin and neuraminidase, which contribute the remaining 97% of total envelope protein. Thus, the total count of protein monomers per virion is about 600. If for the sake of simplicity assume that hemagglutinin homotrimers and neuraminidase homotetramers contribute equally and the average diameter per homo-oligomer transmembrane domain is of the order of 40 Å, we estimate some decrease of the M2-to-lipid molar ratio to about 1:4,000 due to lipid-excluded volume occupied by the embedded proteins. If we further consider the presence of cholesterol of up to ca. 50 mol%^43^, which would also constitute an excluded volume, since M2 does not reside in cholesterol-rich domains, the effective M2-to-lipid molar ratio in the viral envelope would be greater than 1:4,000. However, since a cholesterol molecule is smaller than a lipid and associates with major envelope proteins, the cholesterol-produced excluded volume would not even double this ratio. Thus, as we can see, the native M2-to-lipid molar ratio is considerably lower than what has been used in the majority of structural and functional studies, i.e. being typically 1:10 to 1:100 for NMR^16^,^17^,^18^,^19^,^20^,^24^, and down to 1:1,500 for thiol-disulfide equilibrium experiments^15^. Thus, the question of how M2 tetramers might assemble to perform their function under the more dilute native conditions with lower protein-to-lipid molar ratios has not been answered in these studies.

We studied by DEER the self-association of M2TMD_21–49_ in DOPC/POPS membrane at pH 5.5 in a broad range of P/L molar ratios from 1:18,800 to 1:160 to better understand the assembly pathway and oligomeric abundance of M2. We found that within this P/L range M2TMD_21–49_ peptide exists in multiple oligomeric states. By analyzing the profile of DEER modulation depth as a function of P/L (Figs 3 and 5, Eq. 1), we identified the order of the oligomers that contribute to the DEER signal. They are monomers, dimers and tetramers. The relative population of each oligomeric species strongly depends on P/L ratios: at the lowest P/L of 1/18,800, dimers are prevalent, whereas at the highest P/L of 1:235 and 1:160 tetramers are mainly present. Based on our results, we propose a cascade mechanism in which M2TMD_21–49_ self-associates in two stages: First it forms a strong dimer (2M↔D), which further associates into a dimer-of dimers that is a M2TMD tetramer (2D↔T) (Fig. 5). This is different from monomer-to-tetramer equilibrium that was previously reported, based on combined analytical centrifugation and fluorescence and CD spectroscopies studies performed on M2TMD in DPC micelles^29^. The discrepancy between this study^28^ and our findings could originate from the different membrane mimetics used. However, in later studies on both DPC micelles- and lipid-reconstituted M2TMD, cross-linked dimeric intermediate were observed using thiol-disulfide exchange^15^,^29^, although the results again were interpreted in terms of cooperative monomer-to-tetramer equilibrium. It should be noted that the idea of a M2TMD channel as a functional and conformational dimer-of-dimers was proposed in earlier studies by magic angle spinning solid state NMR^11^,^17^,^52^. However, there was no information on how this dimer-of-dimers might assemble. Here we provide unambiguous structural evidence that in the lipid bilayer M2TMD assembles via a quite stable dimeric intermediate.

Furthermore, our results indicate that M2TMD_21–49_ forms tight dimers at both acidic and basic pH of 5.5 and 8, respectively, since monomers were barely populated even at the lowest P/L used in our experiments (Fig. 5a,b lower panels), being marginal at pH 8. This is supported by the estimated *k*_2d_ (lipid) = 15·10^−6^ MF (~1:65,000 P/L) at pH 5.5 and a very small *k*_2d_ (lipid) at pH 8. What is more, we found that at P/L ratios in the range of native conditions, specifically for M2-to-lipid molar ratios between 1:6,300 and 1:4,000, M2TMD_21–49_ is significantly dimeric, based on the estimated *k*_4d_ (lipid) = 448·10^−6^ MF (~1:2,230 P/L) at pH 5.5 and *k*_4d_ (lipid) = 86·10^−6^ MF (~1:11,600 P/L) at pH 8. Strikingly, our results show that, under the same conditions, the tetramer is less abundant at pH 5.5 than at pH 8. However, at native conditions, M2 is active in the low pH (5 to 6) environment of host cell endosomes, functioning as a proton channel. Therefore, the equilibrium at pH 8 is hardly relevant as M2 is internalized into the lumen of the endosome in a process that takes enough time that is likely more than sufficient to shift the equilibrium. Hence, the possibility that M2 dimers are abundant under native conditions seems very likely. On the other hand, the full-length M2 has two cysteine residues at positions 17 and 19 (C17 and C19), which in most influenza A strains are cross-linked and stabilize the protein tetrameric form^3^. Consequently, the disulfide bond stabilization might change the energy landscape of the full-length M2 assembly with respect to those of truncated M2TMD, resulting in an equilibrium shifted towards tetramers. But it is obvious that such cross-linking is more in line with dimer-of-dimers configuration and eliminates monomers altogether. In addition, it was also demonstrated by mutating out C17 and C19 that M2 does form non-covalently associated oligomers, and cysteine cross-linking is thus not essential for tetrameric oligomerization^3^ as one would reasonably expect. Furthermore, it was suggested in a very recent study that the minimal functional unit of M2 is a dimer based on combining fluorescence resonance energy transfer with coiled-coil tag probe labeling method applied to full-length M2 expressed in the plasma membrane of CHO-K1 cells^32^. Thus, our findings about the existence and high abundance of M2 dimer under P/L ratios close to native, are consistent with the results of these others^32^, and open a new question about M2 native forms. It may well be that an M2 dimer is merely an intermediate step in the M2 channel quaternary structure formation. However, since M2TMD tetramer does form functional channels^12^, it would be interesting in the future to test if significant proton conductance could be detected at P/L ratios less than 1:20,000 where, as our results suggest (Fig. 5), the dimeric form is prevalent. Indeed, after reconstructing the inter-spin distances from DEER time-domain signals, we observed a shift to longer distances upon decreasing pH from 8 to 5.5 under all P/L conditions, including these where the dimer is prevalent (P/L’s higher than1:4,100). This could be an evidence for restructuring also in the dimeric species in response to pH change. The question of whether this conformational transition is related to proton channel type activity of M2 dimer is yet to be answered by future studies. Nevertheless, at present, the possible proton conductance by M2 dimer is rather speculative and dimers may play another functional role, given that similar systems such as the Bnip3 transmembrane domain, which has a histidine residue and forms dimers in membranes^53^ , does exhibit proton conductance. Another possibility may be that the M2 dimer-tetramer equilibrium adjusts as M2 finds itself in membranes of different composition and thicknesses encountered on the pathway from secretion to virus budding and maturation. Thus, active and non-active conductive forms or forms with diverse activities may be regulated by such a simple mechanism, potentially making this protein a very efficient multifunction component of influenza proteome, and this is worthy of future study.

We also found that the equilibrium and consequently the relative binding energies of M2TMD_21–49_ oligomerization in detergent are significantly different from that in lipid: the dissociation constant *k*_2d_ (detergent) is about an order of magnitude greater than that in lipid and is the same order of magnitude as *k*_4d_ (detergent). This is not surprising, as the physicochemical and mechanical properties of detergent micelles are vastly different from that of a lipid bilayer, so the micelles may not provide optimal coupling to accommodate the proteins, whereas the biological membranes provide proper conditions for protein assembly and function.

Our study also demonstrates the versatility of pulse dipolar ESR spectroscopy, specifically Ku-band DEER, which enabled the investigation of a membrane protein assembly in a lipid bilayer environment by ensuring a very high spectral sensitivity which was required to detect oligomers in as low as 5 μM average (bulk) concentrations of M2TMD_21–49_, monomer, for a P/L of 1:18,800. We developed and used a methodology for identifying and analyzing in detail the more complex equilibrium of multiple protein oligomeric species that co-exist in the membrane milieu, and which should generally be useful to study protein self-association and function.

## Methods

### M2TMD_21–49_ synthesis, solubilization and spin-labeling

M2TMD_21–49_ peptide (dssdplvvaasiigilhlilwildr**C**ffk, L46C) was commercially synthesized with higher than 98% purity (GenScript, Inc.) and received as lyophilized powder. The dry peptide was solubilized in solution of *n*-Dodecyl β-D-maltoside (β-DDM) buffered with 25 mM NaPi pH 7.4 and 150 mM NaCl. To ensure the reduced form of cysteine residue, peptide was incubated with agarose beads-immobilized Tris(2-carboxyethyl)phosphine (TCEP) (Thermo Fisher Scientific, Inc.). Next, M2TMD_21–49_ at concentration 120 μM was spin-labeled with S-(2,2,5,5-tetramethyl-2,5-dihydro-1H-pyrrol-3-yl)methyl-methanesulfonothioate (MTSL; Toronto Research Chemicals) following the protocol from Georgieva *et al.*^39^. The unreacted MTSL was removed by dialysis. In the last dialysis step, the peptide solution was divided into two aliquots, which were dialyzed against either 25 mM sodium phosphate (NaPi) pH 8, 150 mM NaCl, and 1 mM β-DDM or 25 mM MES pH 5.5, 150 mM NaCl, and 1 mM β-DDM. Dialysis membrane with 1 kDa MWCF (Spectrum Laboratories, Inc) was used. The final concentration of peptide was 100 μM, estimated using a calculated extinction coefficient of 5,600 M^−1^cm^−1^ and absorbance at 280 nm.

### Preparation of model lipid membranes

Model lipid membranes were prepared of 1,2-dioleoyl-*sn*-glycero-3-phosphocholine (di-18:1 PC or DOPC), 1-palmitoyl-2-oleoyl-*sn*-glycero-3-phospho-L-serine (16:0/18:1 PS or POPS) (Avanti Polar Lipids) at 85:15% molar ratio. First, chloroform solutions of lipid mixtures were dried with a stream of nitrogen gas, and then on a vacuum line for at least 4 h. The dried lipid films were resuspended in buffers of 150 mM NaCl, 1 mM EDTA and 25 mM MES pH 5.5 or 150 mM NaCl, 1 mM EDTA and 25 mM NaPi pH 8 to a final 30 mM total lipid concentration, and kept for 1 h at 4 °C to achieve full hydration.

### Reconstitution of M2TMD_21-49_ in lipid membranes and DEER samples preparation

A standard protocol for lipid reconstitution of membrane proteins^39^ was followed. Multiple samples with a progression in the peptide-to-lipid (P/L) molar ratio were prepared: at P/L of 1:160, 1:235, 1:500, 1:820, 1:1,300, 1:1,650, 1:2,300, 1:4,100, 1:9,400 and 1:18,800 at pHs 5.5 and 8, and an additional samples at P/L 1:200, 1:5,600, at pH 5 and 1:3,170 at pH 8. The sample at P/L 1:18,800 at both pH’s had 80% deuterated buffer. After reconstituting the peptide in lipid, as a final step, all samples were concentrated in microcentrifuge concentrators with 10 kDa MWCF (GE Healthcare Bio-Sciences).

Additional batch of M2TMD_21-49_ peptide in β-DDM at peptide-to-detergent (P/D) molar ratios of 1:10, 1:33, 1:280, 1:660, 1:2,000 and 1:6,000 at pH 5.5 was prepared by diluting the 1:10 stock with required amount of detergent. After dilution all samples were incubated for 2 h before loading ~20 μL amounts into ESR sample tubes followed by flash-freezing in liquid nitrogen for DEER measurements. The final samples contained glycerol as cryoprotectant, 20% (w/v) for lipid and 30% (w/v) for detergent. Glycerol was previously used in DEER studies of membrane proteins, and no effect on protein structure was noticed^54^.

To test the reproducibility of our results, duplicates or triplicates at several P/L ratios at both pH 5.5 and pH 8 were prepared from stock solutions of detergent-solubilized M2TMD_21–49_ at either 100 μM or 74.6 μM and the modulation depths of their DEER signals were compared. The results were found to be highly reproducible (Supplementary Fig. 5).

Since there is always a concern regarding the potential of sample freezing to change the property of the system, such as affect the structure or shift equilibria, this at least needs to be tested. Most of samples were plunge-frozen in liquid N_2_ as described above. Therefore, we tested if the cooling rate of freeze-punch in LN_2_ might have an effect on DEER modulation depth, hence on M2TMD_21-49_ assembly on which it reports. Specifically, after recording the DEER signal from, the sample of P/L 1:3,170 at pH 8 plunge-frozen in liquid nitrogen it was brought up at room temperature, equilibrated, and frozen again much faster by quenching it in *n*-pentane bath cooled down to just above its m.p. at 143 K, and the DEER signal was re-recorded. The *n*-pentane bath is used for rapid-freezing of biological samples and is comparable to isopentane bath. Such an approach ensured for example the preservation (trapping) of gramicidin A head-to-head dimer form which is abundant in L_Α_ phase of DPPC lipid bilayers but depopulated in gel phase^55^. In our case both ways of freezing yielded virtually indistinguishable DEER signals (Supplementary Fig. 6) indicating no measurable effect of large change in freezing rate on M2TMD_21–49_ assembly. It should be noted that in our previous study on ultrafast freezing of soluble globular protein only very minor effects on protein were noticed over 6 orders of magnitude range of freezing rates.

### Pulse ESR measurements

All DEER measurements were performed at 60 K as previously described^39^,^54^ using a 17.3 GHz home-built Ku-band pulse EPR spectrometer^56^. The standard four-pulse DEER sequence^57^ with π/2-π-π pulse widths of 16 ns, 32 ns and 32 ns, respectively, and a 32 ns π pump pulse was used throughout all measurements. A 32 ns pump π-pulse was found optimal for the case of more than two coupled spins^39^. The frequency separation between detection and pump pulses was 70 MHz. The detection pulses were positioned at the low-field edge of the nitroxide spectrum. Typical dipolar evolutions times were 1.4–2 μs as needed with signal averaging lasting from typical 2–4 h up to 24 h for vary high L/P ratios (9,000 to 18,000). The signal background of the raw DEER data was approximated on the semi logarithmic scale by a second or first degree polynomial for lipid and detergent samples, respectively, and subtracted out. (Supplementary Fig. 1). To test the error introduced in the procedure of background subtraction, we varied baseline fitting within reasonable range, leading to estimated error margins of ±2.5% for the smallest P/Ls, which produced the DEER signals with lower signal-to-noise ratio then in remaining cases, where errors were even less. (Supplementary Fig. 7). However, taking into account all typical uncertainties introduced in the course of sample preparation and high but finite reproducibility of conditions and outcome of DEER experiment, we accepted more conservative error margins of ±5%. DQC measurements conducted at 60 K were employed to examine if short distances are present. The six-pulse DQC sequence used π/2 and π-pulses of 3 and 6 ns respectively. The outcome is shown in Supplementary Figure 9c.

### Continuous-wave ESR spectroscopy

The cw-ESR measurements were performed at room temperature (RT), 296 K or low temperature, 163 K, on a Bruker ELEXIS E500 (Bruker, Billerica, USA) spectrometer equipped with a Bruker ER 4122SHQE resonator and Bruker VT-31 temperature controller. For the RT measurements the microwave power used was 1.26 mW and the modulation amplitude was 2.2 G. Low-temperature spectra were recorded at 0.05 mW microwave power and modulation amplitude of 2 G.

### Concentration profile analysis

The modulation depths for each protein concentration given by its MF were obtained from the respective DEER data and the sets of experimental data points were fit to the model based on the equilibrium between monomer, dimer and tetramer using Eq. 1 for the response, which in this case is the calculated modulation depth. Care was taken to accurately account for the parameters affecting the response. Phase relaxation time variations among samples were found to be insignificant (Supplementary Fig. 9a). The modulation depth for the dimer is known within 5% of the theoretical value for the pulse sequence used by calibration and confirmed by numerous experiments conducted in the prior measurements^39^,^46^ on efficiently labeled proteins and organic biradicals. Custom made sample tubes ensured reproducible resonator tuning and the sample height was slightly elevated to ensure its reproducible positioning. For tetramers maximum theoretical modulation depth was calculated according to the literature taking into account the fact that spin-labeling efficiency, *f*, was at least 0.95 for pH 5.5 samples and at least 0.9 for pH 8 samples. This will only weakly affect the depth which is still ~0.5 for f = 0.9. More significantly for 4 spins there will be further reduction of modulation depth due to limited homogeneity of *B*_1_ component of microwave field within the reporting part of sample. This was estimated as capable of ~10% depth reduction. Therefore, one should expect the depth within the range of 0.45–0.5 for tetramers with experimental setup used^36^. All these contribution to *x*_*i*_ of Eq. 1 were included in the data fitting. Modulation depth profiles were fitted in a MATLAB^®^ script using a non-linear least square algorithm implemented as lsqnonlin.m function in optimization toolbox. The constants *k*_2d_ and *k*_4d_ were the fitted parameters. The uncertainty in estimates of experimental parameters *x*_*i*_ and from other estimated experimental errors would result in a reasonable error, estimated as ±50% for the constants. A non-negative constraint was enforced to avoid problems with finding a single positive root of the 4^th^ order algebraic equation for monomer equilibrium concentration from which all other concentrations were calculated. Note that strong dimers found for pH 8 samples practically exclude the possibility of trimers, as monomer concentration becomes quite low at the onset of the concentration range where tetramers form. This observation propagates well to pH 5.5 samples. In addition there are other experimental data and arguments (Discussion) that collectively tend to rule out the existence of trimers entirely.

## Additional Information

**How to cite this article**: Georgieva, E. R. *et al.* Mechanism of influenza A M2 transmembrane domain assembly in lipid membranes. *Sci. Rep.* **5**, 11757; doi: 10.1038/srep11757 (2015).

## Supplementary Material

Supplementary Information
