# Supplementary material for: Mechanism of influenza A M2 transmembrane domain assembly in lipid membranes
Source: Sci Rep. 2015 Jul 20;5:11757. doi: 10.1038/srep11757 (PMC4507135; doi:10.1038/srep11757)
Supplement: Supplementary Information [file srep11757-s1.pdf]

Supplementary Information

Mechanism of influenza A M2 transmembrane domain assembly in lipid membranes

Elka R. Georgieva[1,2*], Peter P. Borbat[1,2], Haley D. Norman[3] and Jack H. Freed[1,2*]

[1] Department of Chemistry and Chemical Biology, [2] National Biomedical Center for Advanced ESR Technology, and [3] School of Engineering, Cornell University, Ithaca, NY 14853

Correspondence should be addressed to erg54@cornell.edu and jhf3@cornell.edu

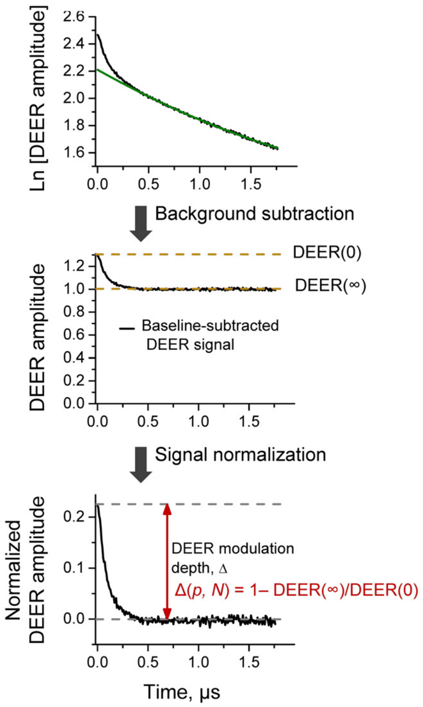

Supplementary Figure 1. DEER data preprocessing and presentation as used in this work.

Supplementary Fig. 1 illustrates DEER data preprocessing and presentation used in this work, given that other procedures are common in utilizing this method. The raw time-domain DEER signal (in black) and the background signal commonly called 'baseline' (in green) are shown on

a semi-logarithmic scale in the upper panel. All DEER data in this study appear as non-oscillating decays, each reaches the asymptotic value, V at t=∞, $V(\infty)$. It is needed to determine the "modulation depth" $\Delta(p, N) \equiv 1-$ DEER(t=∞)/DEER(t=0) defined in the main text and designated as $\Delta(p, N) \equiv 1-$ DEER(∞)/DEER(0) in the figure. After fitting the baseline to a straight line or a 2nd degree polynomial, the background-subtracted DEER signal is plotted on a linear scale in the middle panel. Now it becomes $V(0) = 1 + \Delta$ and $V(\infty) = 1$. After subtracting unity and normalization by dividing by $V(0)$ it finally appears in the lowest panel such that $V(0)$ is $\Delta(p, N)$ and $V(\infty) = 0$. Thus, the modulation depth, Δ, can be read directly from the plot and is used in this work to estimate the number of interacting spins in a nano-object based on known approaches [1-4].

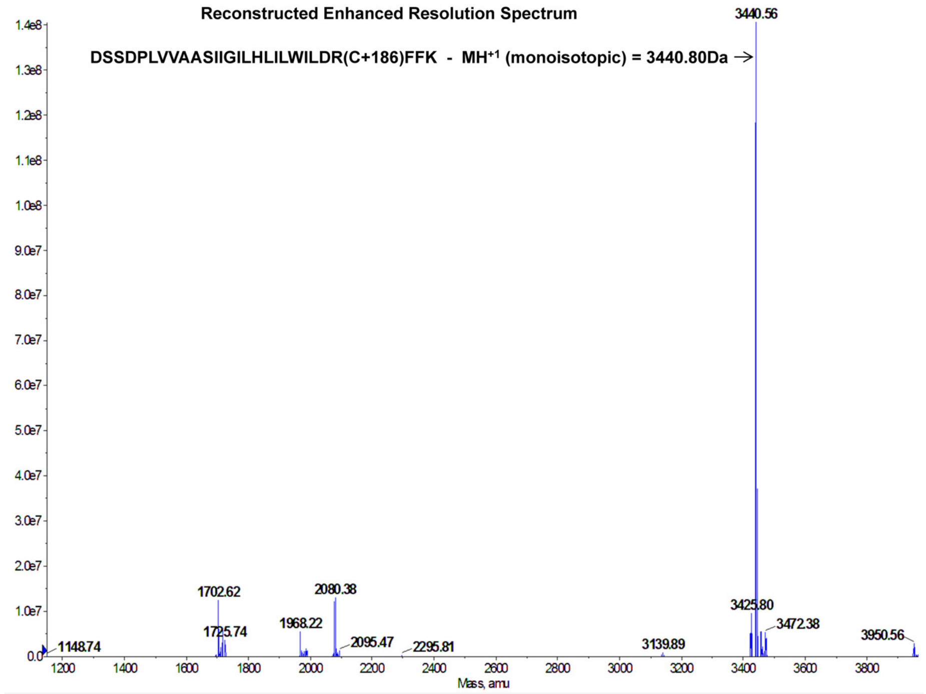

Supplementary Figure 2. Mass spectrum shows close to 100% (>95%) spin-labeling efficiency of $M2TMD_{21-49}$ construct (M2TMD), based on the experimentally obtained molecular weight, which for M2TMD singly spin-labeled with MTSL is 3440.80 Da

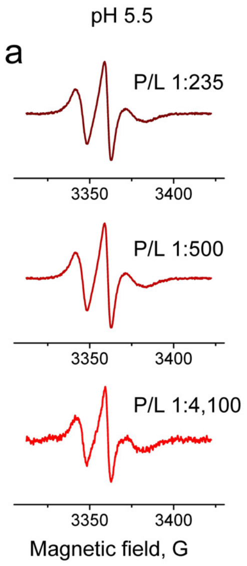

Supplementary Figure 3. Room temperature (23 °C) cw-ESR spectra for spin-labeled L46C residue in M2TMD$_{21-49}$ residing in DOPC/POPS membranes. **a** Low pH data, pH 5.5, for P/L of 1:235, 1:500 and 1:4,100 are shown. **b** Data taken at pH 5.5 and pH 8 are compared for P/L of 1:235. The cw-ESR spectra are all similar indicating very similar dynamic properties with rotational correlation time, τ_c, in the nanosecond range. No contribution from free spin-label to the recorded cw-ESR spectra could be noticed.

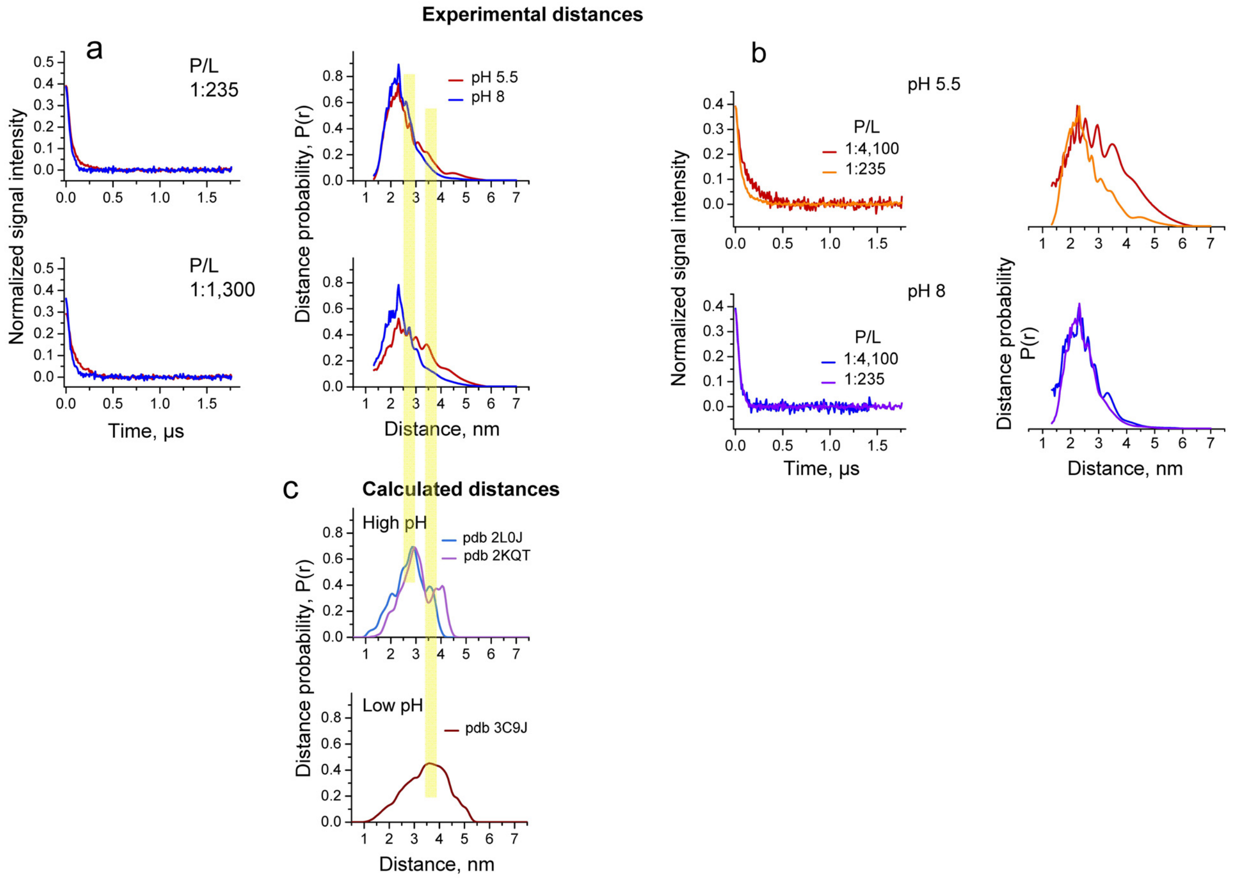

Supplementary Figure 4. **a** A pH-induced effect on M2TMD$_{21\text{-}49}$ assembly is demonstrated for M2TMD$_{21\text{-}49}$ reconstituted in DOPC/POPS membranes at peptide-to-lipid ratios (P/L) of 1:235 and 1:1,300 and at pH's 5.5 (red) and 8 (blue).

b The dependence on P/L of DEER time-domain signals (left) and corresponding distances (right) at both pH 5.5 and pH 8 is demonstrated by comparing the data for P/L's of 1:235 and 1:4,100. In all cases the DEER data and distance distributions are normalized to have the same intensity. In both **a** and **b**, the experimental time-domain DEER data are shown on the left and respective distance distributions are on the right. The distance distributions in these plots were reconstructed using Tikhonov regularization [5] and refined by the Maximum Entropy Method [6].

c The distance distributions generated using Molecular Modeling Software (MMM) [7] based on high pH (pH 7.5) NMR structures in DOPC/DOPE (pdb ID 2L0J) [8] and in DMPC with amantadine drug bound in M2 channel (pdb ID 2KQT) [9] are shown in the upper panel. The

calculated distance distribution based on the x-ray crystal structure of detergent-solubilized $M2TMD_{21\text{-}49}$ at pH 5.3 (pdb ID 3C9J) [10] is shown in the lower panel.

Overall, we find good agreement between calculated and experimental distances. However, in most cases our distance distributions are produced by co-existing $M2TMD_{21\text{-}49}$ dimers and tetramers with their contributions depending on P/L ratio. In general, dimers contribute just one distance, whereas for a tetramer, both laterally and proximally distant spins define the DEER signal and distances. In the case of a homotetramer with 4-fold symmetry two distances r_2 and $r_1 = 2^{1/2}r_2$ with populations in ratio 2:1 could be reconstructed from the time-domain DEER signal, as previously discussed [11]. (r_1 and r_2 here denote proximal and lateral distances, respectively). In the relevant case of a lower symmetry or for an asymmetric tetramer more distances (up to 6) may be present, resulting in a less tractable case.

It is noteworthy that we observed an increased population of longer distances in the range of 2.5-3.5 nm when lowering the P/L, i.e. increasing the population of $M2TMD_{21\text{-}49}$ dimers.

Another trend is a shift to longer distances upon decreasing pH from 8 to 5.5, that was observed for all P/L conditions, including those where dimer is prevalent (P/L's higher than 1:4,100), thus indicating that the dimer also experiences pH sensitive conformational changes.

In summary, we did observe a conspicuous pH-induced shift to longer distances upon decreasing the pH from 8 to 5.5, which might be reasonably attributed to M2TMD channel opening. However, investigating the details of this structural transition is outside the focus of the present study.

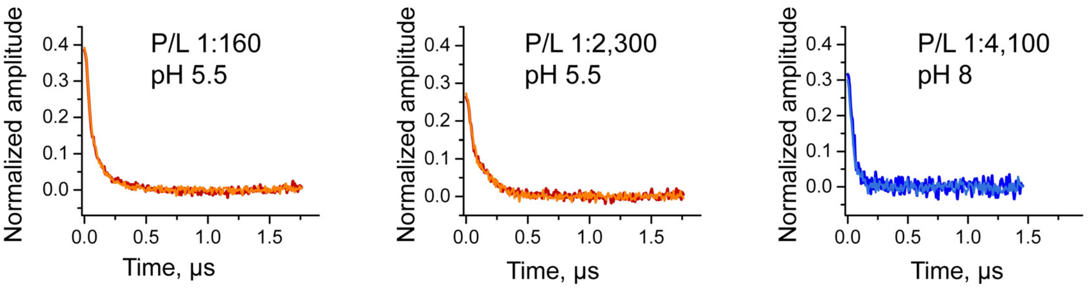

Supplementary Figure 5. High degree of DEER data reproducibility for independent sample preparations is illustrated for selected typical cases, P/L 1:160 and 1:2,300 at pH 5.5, and 1:4,100 at pH 8. Data for duplicate samples from different preparations are superimposed in each panel. In all cases the extent of data variations is negligible.

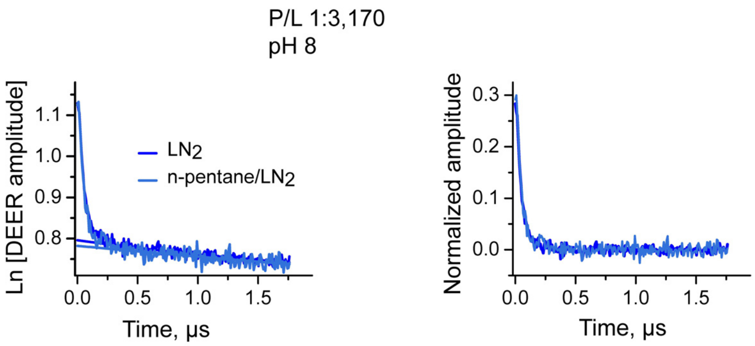

Supplementary Figure 6. The lack of effect of sample freezing rate on the DEER modulation depth for M2TMD$_{21\text{-}49}$ spin-labeled at position L46C reconstituted into DOPC/POPS membranes at pH 8 and P/L 1:3,170.

Data for samples plunge-frozen in liquid nitrogen or much faster by quenching in *n*-pentane kept just above its melting point of -130 ºC, are shown in shades of blue. The raw DEER data rendered on semi-logarithmic scale together with the corresponding second-order polynomial baselines and the background-subtracted DEER data are plotted in the left and right panels, respectively. For these two freezing methods, we found only very minor difference ~0.01 in the DEER modulation depths, which is almost within the level of uncertainty encountered in fitting

the baselines. Moreover, no change of the signal shape exceeding the noise level was detected. This indicates that it is unlikely that in our procedures there could be significant effects of lipid freezing on the $M2TMD_{21-49}$ state and its assembly in particular. This is in line with observing just minor effects in freezing of a globular soluble protein [12].

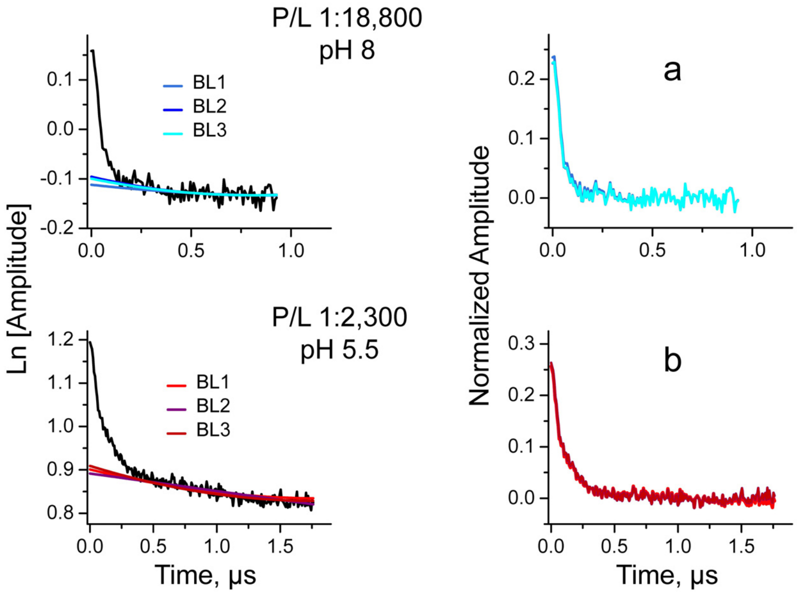

Supplementary Figure 7. DEER modulation depth variations that are too small to be significant are produced due to uncertainties in background fitting.

The data shown in Supplementary Fig. 7 were taken for $M2TMD_{21-49}$ in DOPC/POPS at 85:15 molar ratio. In the left panels, the raw DEER data (in black) are plotted on a semi-logarithmic scale together with manually varied baselines (BL1-BL3) rendered in shades of blue and cyan for pH 8 and in shades of red for pH 5.5. The background-subtracted and normalized DEER data presented in the right panels are rendered in colors corresponding to those of the baselines used in the subtractions. The data for sample with P/L 1:18,800 at pH 8 are shown in row **a**, whereas the data for sample with P/L 1:2,300 at pH 5.5 are shown in row **b**.

The baselines for pH 8 were fit to a second order polynomial on semi-logarithmic scale. In the case of pH 5.5, linear baseline (plotted in violet) was also adequate. Using the approach

outlined, we estimated an error due to uncertainty in background subtraction not to exceed ±2.5% for all processed DEER data.

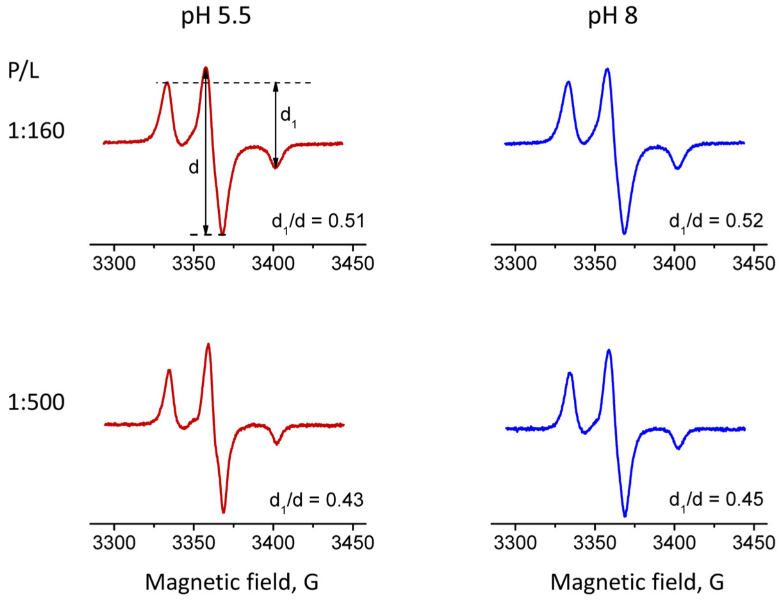

Supplementary Figure 8. Low-temperature (163 K) cw-ESR spectra for spin-labeled L46C residue in $M2TMD_{21\text{-}49}$ in DOPC/POPS membranes. Data for pH 5.5 and pH 8 at P/Ls of 1:160 and 1:500 are shown.

To examine the possible presence of short distances (shorter than is in the sensitivity range of DEER, i.e. less than 1.7 nm), we recorded the cw-ESR spectra of $M2TMD_{21\text{-}49}$ frozen samples. Finding the d_1/d parameter from the cw-ESR spectra of frozen samples, as illustrated by Supplementary Fig. 8, is a commonly used empirical method for estimating strong dipole-dipole interactions arising at shorter than 2.0 nm distances between paramagnetic centers [13]. According to this method an inter-spin distance of 1.89 nm corresponds to d_1/d = 0.54; further increase in the inter-spin distances leads to smaller values for the d_1/d parameter. In our study, we measured a d_1/d parameter of less than 0.54, indicating the presence of distances, which are well within the sensitivity range of DEER experiment. Furthermore, the largest d_1/d values were obtained for P/L of 1:160 for both pH 5.5 and pH 8. These values gradually decrease with the increase of P/L (P/L 1:160 vs. P/L 1:500 in the figure), i.e. when reducing the population of $M2TMD_{21\text{-}49}$

tetramer. Hence, most likely, only the M2TMD$_{21\text{-}49}$ tetramer has a distance in the range of 2.0 nm or slightly less, which also agrees with our data in Supplementary Fig. 4.

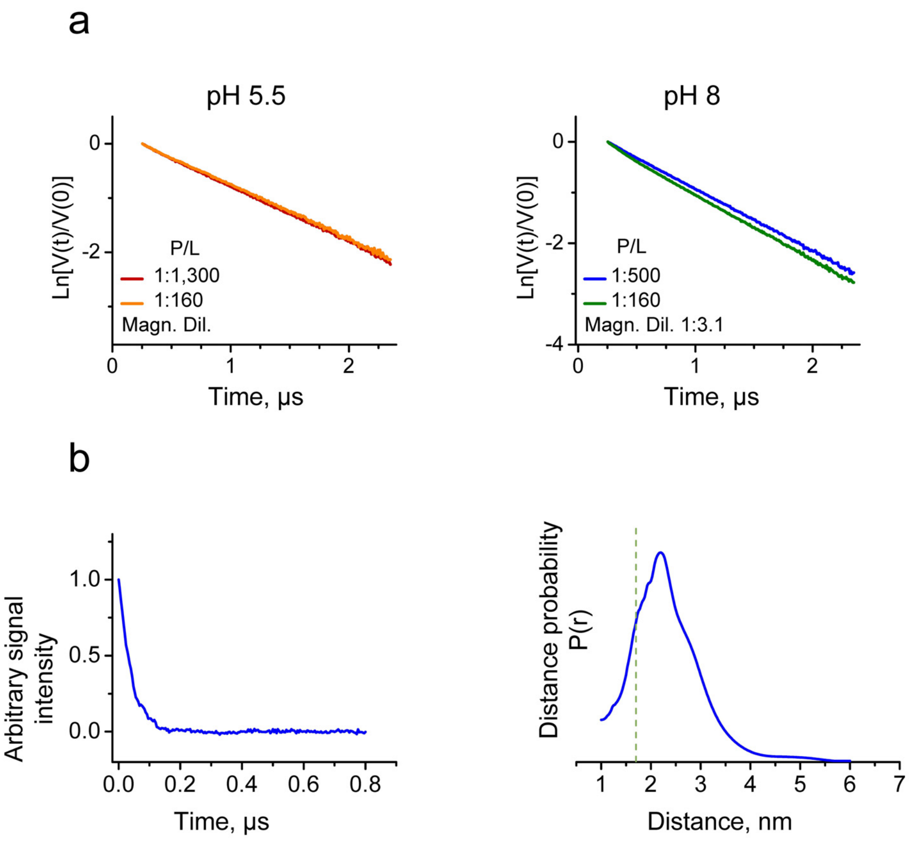

Supplementary Figure 9. a Primary spin-echo echo decays for samples spin-labeled at position L46C in M2TMD$_{21\text{-}49}$ are shown. Data plotted on the left were taken at pH 5.5 for P/L of 1:1,300 and for P/L of 1:160, but for peptide magnetically diluted with non-labeled peptide to about the same number of spins. Data on the right were taken at pH 8 for P/L of 1:500 and for P/L of 1:160, but with peptide magnetically diluted with non-labeled peptide to about the same number of spins. **b** Time-domain double-quantum coherence (DQC) signal (left) and reconstructed distances (right) for the magnetically diluted sample at pH 8. The time-domain DQC was normalized to unity at zero evolution time.

To collect the primary spin-echo decay data, a standard π/2-π pulse sequence with 250 ns initial inter-pulse separation was used.

Within the accuracy of relaxation experiment, our data for non-magnetically diluted and magnetically diluted samples at pH 5.5 are nearly identical. Only a minor difference is noticed for pH 8. Thus, the primary spin-echo decays due to phase memory relaxation, at P/L ratio of 1:160, where tetramers are dominant species, are very close to those where dimers are also significantly present, i.e. P/L 1:1,300 and 1:500.

The DQC data indicate that at most there is only a very small population of distances shorter than 1.7 nm (green dashed line). Given that there is usually a broadening ("wings") of distance distribution due to noise-dependent stabilization properties of Tikhonov regularization, the fraction of spin-pairs at distances less than 2.0 nm is likely even less than what is seen in panel **c**.

Supplementary References

1. Milov, A.D., Ponomarev, A.B. & Tsvetkov, Y.D. Electron-electron double resonance in electron spin echo: Model biradical systems and the sensitized photolysis of decalin. *Chemical Physics Letters* **110**, 67-72 (1984).

2. Bode, B.E. et al. Counting the monomers in nanometer-sized oligomers by pulsed electron-electron double resonance. *J Am Chem Soc* **129**, 6736-45 (2007).

3. Borbat, P.P. & Freed, J.H. Pulse Dipolar ESR: Distance Measurements. in *Structure and Bonding* 1-82. (Springer, Heidelberg 2014).

4. Jeschke, G., Sajid, M., Schulte, M. & Godt, A. Three-spin correlations in double electron-electron resonance. *Phys Chem Chem Phys* **11**, 6580-91 (2009).

5. Chiang, Y.W., Borbat, P.P. & Freed, J.H. The determination of pair distance distributions by pulsed ESR using Tikhonov regularization. *J Magn Reson* **172**, 279-95 (2005).

6. Chiang, Y.W., Borbat, P.P. & Freed, J.H. Maximum entropy: a complement to Tikhonov regularization for determination of pair distance distributions by pulsed ESR. *J Magn Reson* **177**, 184-96 (2005).

7. Polyhach, Y., Bordignon, E. & Jeschke, G. Rotamer libraries of spin labelled cysteines for protein studies. *Phys Chem Chem Phys* **13**, 2356-66 (2011).

8. Sharma, M. et al. Insight into the mechanism of the influenza A proton channel from a structure in a lipid bilayer. *Science* **330**, 509-12 (2010).

9. Cady, S.D. et al. Structure of the amantadine binding site of influenza M2 proton channels in lipid bilayers. *Nature* **463**, 689-92 (2010).

10. Stouffer, A.L. et al. Structural basis for the function and inhibition of an influenza virus proton channel. *Nature* **451**, 596-9 (2008).

11. Dalmas, O., Hyde, H.C., Hulse, R.E. & Perozo, E. Symmetry-constrained analysis of pulsed double electron-electron resonance (DEER) spectroscopy reveals the dynamic nature of the KcsA activation gate. *J Am Chem Soc* **134**, 16360-9 (2012).

12. Georgieva, E.R. et al. Effect of freezing conditions on distances and their distributions derived from Double Electron Electron Resonance (DEER): a study of doubly-spin-labeled T4 lysozyme. *J Magn Reson* **216**, 69-77 (2012).

13. Kokorin, A.I. Forty years of the d1/d parameter. (ed. Kokorin, A.I.) (InTech, 2012, p. 113-164).
